# Ligament-Derived Stem Cells: Identification, Characterisation, and Therapeutic Application

**DOI:** 10.1155/2017/1919845

**Published:** 2017-03-12

**Authors:** Katie Joanna Lee, Peter David Clegg, Eithne Josephine Comerford, Elizabeth Gail Canty-Laird

**Affiliations:** ^1^Department of Musculoskeletal Biology, Institute of Ageing and Chronic Disease, University of Liverpool, William Henry Duncan Building, 6 West Derby Street, Liverpool L7 8TX, UK; ^2^School of Veterinary Science, University of Liverpool, Leahurst Campus, Chester High Road, Neston CH64 7TE, UK; ^3^The MRC-Arthritis Research UK Centre for Integrated Research into Musculoskeletal Ageing (CIMA), Liverpool, UK

## Abstract

Ligament is prone to injury and degeneration and has poor healing potential and, with currently ineffective treatment strategies, stem cell therapies may provide an exciting new treatment option. Ligament-derived stem cell (LDSC) populations have been isolated from a number of different ligament types with the majority of studies focussing on periodontal ligament. To date, only a few studies have investigated LDSC populations in other types of ligament, for example, intra-articular ligaments; however, this now appears to be a developing field. This literature review aims to summarise the current information on nondental LDSCs including* in vitro* characteristics of LDSCs and their therapeutic potential. The stem cell niche has been shown to be vital for stem cell survival and function in a number of different physiological systems; therefore, the LDSC niche may have an impact on LDSC phenotype. The role of the LDSC niche on LDSC viability and function will be discussed as well as the therapeutic potential of LDSC niche modulation.

## 1. Introduction

Ligament is prone to injury and degeneration, particularly the anterior cruciate ligament (ACL) [1], with an incidence of approximately 37 ACL ruptures per 100,000 people [2] and a greater incidence among athletes [3]. Healing after ligament injury is poor leaving abnormal scar tissue which frequently is unable to function effectively [4]. The current treatment strategies for ligament injury are limited with variable success rates. Rest and physiotherapy are often prescribed along with a knee brace for ACL injuries to aid stability of the knee [5]. In more severe cases or where conservative therapies have failed, surgery is often performed; however, there appears to be little difference in success outcomes between surgical and conservative treatment options [6]. Most cases requiring surgery for ACL ruptures undergo reconstruction of the ligament using a section of the patients' hamstring, or patellar tendon, or, less commonly, allogeneic grafts. There are variable success rates associated with ACL reconstruction, dependent upon the patient's lifestyle, age, and health [6, 7]; however, recent advances in the field of stem cell research may provide a treatment option with improved success rates. For example, the injection of mesenchymal stem cells (MSCs) alone [8] or with the use of a biosynthetic scaffold [9] to treat ACL rupture shows promising results at the preclinical research stage. It is clear that stem cell therapies such as MSCs hold potential for treatment of ligament injuries and the identification of stem cells in ligament tissue [10] may also provide a possible therapeutic option.

In 2004, Seo et al. identified a population of cells within periodontal ligament which demonstrated certain MSC characteristics, including clonogenicity, expression of stem cell markers, and the ability to differentiate down a number of different cell lineages [10]. Since then a large amount of research has been conducted into periodontal ligament stem cells (PDLSCs), both into the characterisation of these cells [10, 11] and into their use in tissue engineering strategies [12, 13]. Therefore, the majority of published research on stem cells in ligament has focussed on PDLSCs. The promising results seen with these cells may also be applicable for other ligaments in other areas of the body, and in recent years research has turned to the ACL and the potential for ligament-derived stem cells (LDSCs) to provide therapies for other types of ligament injury. This literature review will focus on the identification, characterisation, and therapeutic potential of LDSCs derived from nondental origins, with particular emphasis on stem cells isolated from the ACL.

## 2. Isolation and Culture of LDSCs

The majority of studies investigating nondental LDSCs have isolated cells from human ACLs (ACLDSCs) [14, 15]. However, there are a number of other studies which have isolated and cultured LDSCs from other species, including horses [16], pigs [17], and rabbits [18], as well as other ligament types, including rabbit medial collateral ligament (MCL) [18] and human interspinous ligament [19].

The isolation of LDSCs involves tissue extraction, digestion in collagenase, and seeding of cells [14, 20] or, alternatively, tissue extraction and outgrowth of cells from ligament explants [21, 22]. Despite the different approaches to LDSC isolation, cells obtained through tissue digestion or tissue explants seem to demonstrate similar stem cell characteristics [22]. Cells are then cultured* in vitro* and can be extensively expanded up to 25 population doublings [21] or 20 passages [16]. Unlike other stem cell types, there is little research on the optimum culture conditions for LDSC survival and expansion. The survival and function of cells are normally dependent upon the culture conditions, and oxygen tension appears to have an effect on LDSC metabolism and matrix production [23]. In addition, certain media formulations and growth factors, such as basic fibroblast growth factor (bFGF) and transforming growth factor *β*1 (TGF-*β*1), promote stem cell proliferation and differentiation [24]. However, the effects of many other factors associated with* in vitro* culturing of cells have yet to be investigated.

## 3. Characteristics of LDSCs

### 3.1. Clonogenic Potential and Cell Morphology of LDSCs

**Figure 1:**
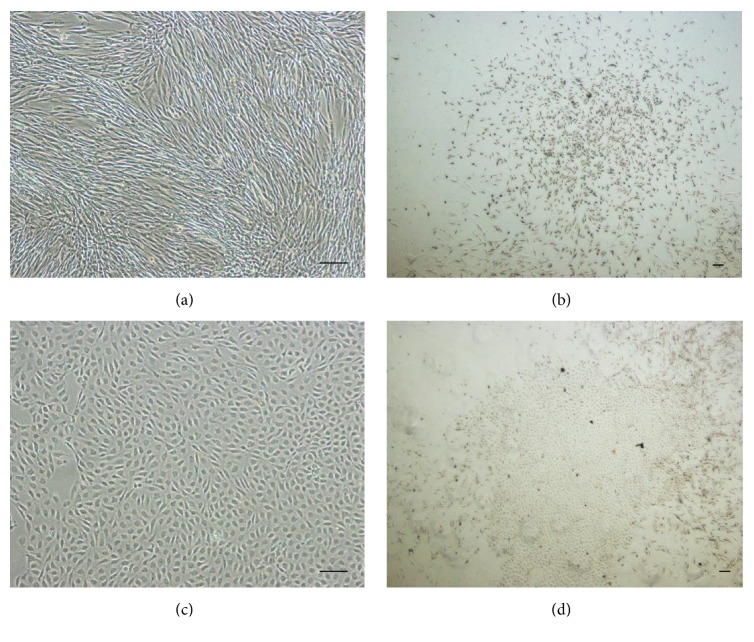
Different morphologies and clonogenic ability of LDSCs isolated from canine cranial cruciate ligament. (a)-(b) show sparse colonies with a fibroblastic cell morphology. (c)-(d) show dense colonies with a rounded, cobblestone cell morphology. Bars = 100 *μ*m.

After several days in culture, the LDSCs start to form heterogeneous colonies [20, 21], reflecting the heterogeneity of cell populations and differences in proliferation rates [21]. The majority of studies have reported a fibroblastic cell morphology of LDSCs [24, 25]; however, there seems to be variation between ligament types. LDSCs isolated from ACL and posterior cruciate ligament (PCL) demonstrated a fibroblastic morphology [15, 21] and those from MCL a polygonal morphology [18, 20]. LDSCs isolated from equine suspensory ligaments (SLs) demonstrated a heterogeneous morphology, with predominantly fibroblastic cells, but also with the presence of smaller rounded cells [16]. We have observed both rounded and fibroblastic cell morphologies upon initial seeding of stem cells derived from canine cranial cruciate ligament (CCL); however, the rounded morphology was lost with passaging (Figure 1).

### 3.2. Stem Cell and Tenogenic Marker Expression of LDSCs

Many of the markers used to identify LDSCs are found in other cell types, including MSCs and tendon-derived stem cells (TDSCs) (Table 1).

Stem cells derived from human ACL tissue have been shown to express the MSC markers CD13, CD29, CD44, CD49c, CD73, CD90, CD97, CD105, CD146, CD166, SSEA-4, STRO-1, and HLA A, B, C, as well as the pluripotency markers Oct4, Nanog, and Sox2, and demonstrate negative expression of CD34 and CD45 [20–22, 25]. Stem cells derived from rabbit MCL have been shown to express CD44 and CD90 but not CD34, CD106, or CD11b [18].

LDSCs have also been shown to express ligamentogenic markers, indicative of their lineage. These markers include the ligament extracellular matrix (ECM) proteins tenascin C [14, 25] and tenomodulin [22].

### 3.3. Multipotency of LDSCs

As a subpopulation of MSCs, LDSCs should be multipotent and have the potential to differentiate into various cell types, including osteogenic, adipogenic, and chondrogenic lineages.

LDSCs isolated from human ACL have been shown to differentiate into osteogenic, adipogenic, and chondrogenic cells. Osteogenesis was confirmed using alizarin red staining for the identification of calcium nodule formation [25, 26] and staining for alkaline phosphatase activity [15, 21]. In addition, the expression of osteogenic marker genes such as osteopontin, osteocalcin, collagen type I, Runx2, and alkaline phosphatase was verified [15, 21]. Adipogenesis was demonstrated using oil red O staining for lipid droplet formation [25, 26], as well as analysis of adipogenic marker gene expression, including FABP4, PPAR*γ*, and LPL [20, 21]. Chondrogenesis was confirmed using alcian blue and toluidine blue staining for glycosaminoglycan formation [25, 26], as well as expression of chondrogenic marker genes including collagen type II, SOX9, and aggrecan [15, 21].

**Figure 2:**
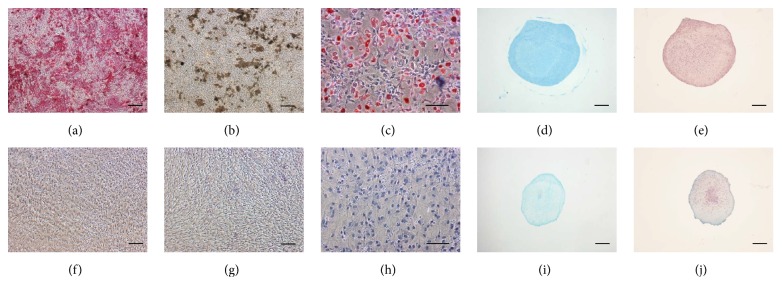
Trilineage differentiation potential of canine cranial cruciate LDSCs. Images are shown for LDSCs after induction of osteogenic, adipogenic, and chondrogenic differentiation after appropriate staining (a)–(e). Cells subjected to osteogenic differentiation media were stained for both calcium deposits using alizarin red stain (a) and alkaline phosphatase activity (b). Cells subjected to adipogenic differentiation media were stained for oil droplet formation using oil red O (c), and cell pellets exposed to chondrogenic differentiation media, for GAG formation using alcian blue (d) and safranin O (e). Negative control cells were incubated in control media and stained as above (f)–(j). Bars = 100 *μ*m.

Osteogenic, adipogenic, and chondrogenic differentiation has also been demonstrated in other ligament types, including LDSCs isolated from equine suspensory [16], human interspinous [19], human medial collateral [20], and human posterior cruciate [21] ligaments, as well as LDSCs derived from canine CCL in our hands (Figure 2).

### 3.4. Variation between LDSCs Isolated from Different Ligaments

There are clear variations in stem cell characteristics of cells isolated from different ligament types; however, there are also some ligaments which share the same cellular properties. LDSCs isolated from human ACL and PCL share the same phenotype, with no discernible or significant differences between the two cell populations [21]. In contrast, there are a number of differences between LDSCs isolated from ACL and MCL, with MCL derived cells demonstrating increased clonogenicity and proliferation rates, increased expression of stem cell marker genes, reduced osteogenesis, and increased chondrogenesis compared with ACL derived cells. In addition, cells isolated from the two ligaments demonstrated differing morphologies with LDSCs from ACL showing a fibroblastic morphology and cells from MCL a polygonal morphology [20]. The possible reasons for these differences have been attributed to the increased vascularity of MCL when compared with ACL, with the subsequent increased healing potential of MCL over ACL [27, 28] potentially being a result of the differences in resident stem cell populations [20]. Another study comparing the differences in stem cell properties between cells isolated from rabbit ACL and MCL found increased chondrogenesis of ACL derived cells compared with MCL derived cells [29], which is in direct contrast to the study discussed above [20]. This discrepancy may be due to ligament cells being seeded at a higher density in the latter study in order to avoid colony formation and therefore their cell population was likely a mixed population of cells with differing properties or, alternatively, species variation may account for the differences [29].

LDSCs are hypothesised to be a subset of MSCs and these two cell populations share many stem cell characteristics, however there are also important differences. LDSCs from human ACL have been directly compared with bone marrow-derived MSCs. LDSCs and MSCs both demonstrate a fibroblastic morphology as well as the ability to form colonies with LDSCs demonstrating increased clonogenicity compared with MSCs; however, proliferation rates seem to vary between studies [14, 22, 25]. Both cell types are able to differentiate into osteogenic, adipogenic, and chondrogenic cell types; however, osteogenic and chondrogenic potential is increased in MSCs compared with LDSCs. In addition, tendon/ligament marker expression is increased in LDSCs compared with MSCs, although stem cell marker expression seems to be consistent across cell types [14, 22, 25].

### 3.5. The Effects of Ageing and Injury on LDSCs

Ageing and injury/degeneration have both been shown to have an effect on the function and phenotype of various stem cell types, including MSCs [30, 31], TDSCs [32–34], and LDSCs isolated from periodontal ligament [35, 36]. However, little is known about the effects of these factors on LDSCs isolated from ACL or other ligament types.

In LDSCs derived from human ACL, age has been shown to cause a decrease in the rate of proliferation as well as reduced osteogenesis [37]. However, another study found few differences between LDSCs isolated from young and old ACL tissue, with a comparable phenotype and multipotency [26].

Stem cells derived from ruptured human ACL remnants and healthy tissue show a similar morphology, clonogenic capacity, stem cell marker expression profile, and multipotency [20, 21]. Further analysis into the effects of ACL rupture on LDSC phenotype showed a decline in LDSC function with increasing time since rupture. LDSCs isolated from acutely injured ACL demonstrated very similar characteristics to those from healthy tissue, whereas cells isolated from chronic injuries showed a reduction in clonogenic potential and multipotency [38].

### 3.6. An Alternative Source of LDSCs

The cells isolated in the aforementioned studies have all been negative for haematopoietic and endothelial markers; however, one group has identified a cell population within the ACL which is positive for CD34 [37, 39], suggesting an alternative vascular source for stem cells located within the ACL. This group identified two populations of cells within ACL tissue: CD34+ and CD34−. CD34+ cells lost expression of CD34 with passaging (whereas expression of MSC markers such as CD44 and STRO1 increased), and this population of cells retained the ability to form endothelial cells to a greater extent than that of CD34− populations [37, 39]. It is likely that LDSC populations are heterogeneous and are in fact a mixed population of cells. The studies mentioned in previous sections may be analysing gene expression at too late a passage to detect CD34 expression. Alternatively, the presence of CD34+ cells within ACL may be a consequence of ACL rupture, as both studies analysed injured tissue. Further investigation in to the phenotype of these differing cell populations and their capacity for ligament repair is warranted.

## 4. The Use of MSCs in Ligament Repair

There is a wealth of literature on the use of MSCs for the repair of ligament, particularly ACL. Studies have used MSCs to repair damaged ACL, as well as using them to enhance healing of ACL tissue after reconstruction surgery.

A variety of approaches have been utilised to aid in repairing of ACL with MSCs. One such technique involved injection of MSCs alone into induced ACL tears in a rat model. The addition of MSCs improved ligament tissue formation and increased ultimate failure load when compared with control animals [8]. Other studies have used biosynthetic scaffolds alongside MSCs to improve ACL repair. The addition of a collagen type I scaffold seeded with MSCs to induced ACL injuries in a rabbit model resulted in improved tissue regeneration with an organised collagen and vascular structure [9]. Silk-based scaffolds have also shown promising results, with MSCs expressing ligament-specific extracellular matrix components and producing ligament-type tissue when implanted with silk scaffolds into a porcine injury model [40].

MSCs have been used to enhance healing of tendon grafts used in ACL reconstruction surgeries. Rabbit Achilles tendons were irradiated and seeded with MSCs before implantation into the ACL site. MSCs increased cellular infiltration and collagen deposition in the repairing tissue [41]. In addition, seeding MSCs on to rabbit hamstring tendon grafts promoted fibrocartilage formation and tendon-bone healing [42].

It is clear that MSCs provide a promising therapeutic option for the treatment of ACL injuries; however, a number of studies have indicated the inferiority of MSCs for treatment of tendon/ligament injuries when compared with native cells isolated from the tissue of interest. Stem cells derived from ligament demonstrate increased clonogenicity and ligament ECM gene expression when compared with bone marrow-derived MSCs (BMMSCs) [14, 25], and collagen scaffolds seeded with tendon cells and implanted into injured tendon showed improved mechanical properties compared with BMMSCs [43]. In addition, stem cells isolated from tendon demonstrate increased clonogenicity, proliferation, multipotency, and stem cell and tenogenic marker expression when compared with BMMSCs [44]. These studies suggest that cells derived from tendon and ligament themselves would ameliorate therapeutic outcomes for tendon and ligament injury when compared with BMMSCs.

## 5. The Use of LDSCs in Ligament Repair

Recent studies have investigated the use of PDLSCs in tissue engineering approaches in dentistry for the repair of periodontal cementum/bone and ligament with successful outcomes [12, 13, 45]; however, little research has been conducted in to the use of LDSCs for other forms of ligament repair.

One study has used rabbit stem cells derived from the MCL in order to repair a MCL injury. When introduced to the injury site, MCL stem cells (MCLSCs) alone induced repair and remodelling with expression of collagen type I and the formation of aligned collagen fibres, as well as increased vascularisation. Functional recovery was also assessed with the use of MCLSCs aiding ligament formation better able to withstand mechanical strain and failure loads. However the addition of CD34+ cells derived from human umbilical cord blood in combination with MCLSCs produced an even greater effect than MCLSCs or CD34+ cells alone. Collagen expression was increased, the fibre alignment scores were higher, vascularisation was increased, and also increased failure loads were tolerated [18]. This study highlights the potential use of LDSCs for repair of ligament injuries outside the field of dentistry. In addition, this study demonstrates that although LDSCs can promote repair of ligament injury, the addition of other cell types allows this healing effect to be increased.

As previously discussed, LDSCs positive for CD34 have also been isolated from ACL tissue and one study has used these cells to investigate the effects of angiogenesis on ACL repair strategies [46]. CD34+ ACLDSCs were transfected with vascular endothelial growth factor (VEGF) and grown in to cell sheets which were wrapped around tendon grafts. These grafts were then implanted into rat joints, in place of previously excised ACLs. The grafts in combination with the cell sheets exhibited increased tensile strength and load to failure when compared with tendon grafts alone. In addition, tendon-bone healing was increased with the use of LDSC sheets, with improved collagen fibre alignment and increased collagen production. The main focus of the study was to assess vascularisation of healing tissue, and with LDSC sheets angiogenesis was significantly increased compared with tendon graft only controls. A VEGF antagonist was also used to show the expected decrease in angiogenesis in the absence of VEGF, which also revealed a decrease in tendon-bone healing [46]. This study demonstrates the potential of vascular-derived LDSCs in treatment strategies for ligament repair as well as the importance of angiogenesis during ligament healing.

## 6. The LDSC Niche

**Figure 3:**
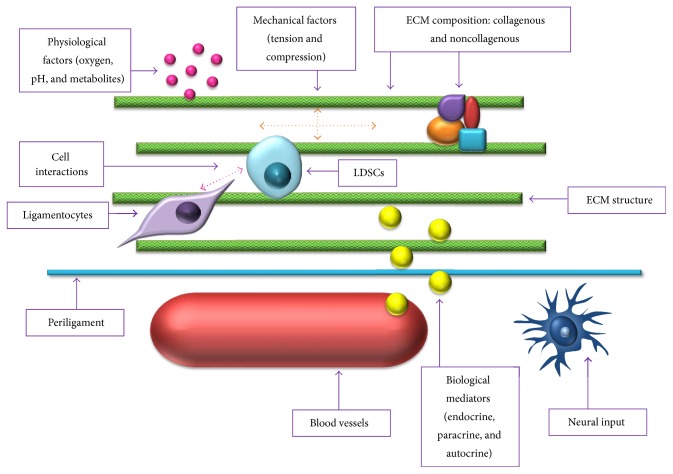
The potential factors comprising the ligament stem cell niche.

There is currently very little known about the LDSC niche and, as with the majority of research focussing on LDSCs, studies investigating the niche have mainly focussed on periodontal ligament; however, there are a few studies which have investigated and identified key components of the niche for nondental LDSCs (Figure 3).

### 6.1. Extracellular Matrix

The composition and structure of the ECM have been found to have significant effects on various stem cells including PDLSCs [12, 47] and TDSCs [48, 49], and so it is likely that the ECM has an impact on LDSC viability and function, although to date no studies have investigated this.

### 6.2. Oxygen Tension

Oxygen tension has been shown to increase metabolism and collagen expression of stem cells isolated from ACL [23].

### 6.3. Biological Mediators

The presence and quantity of certain biological mediators also have an impact on LDSC properties. For ACLDSCs, certain media formulations and the presence of specific growth factors, for example, FGF and TGF-*β*1, influence various stem cell properties, including proliferation and multipotency [24]. ACLDSCs transfected with BMP-12 and BMP-13 demonstrate increased ECM production [50], indicating the likely importance of these mediators in tissue homeostasis.

### 6.4. Cell Interactions

Although no studies have investigated the link between ligamentocytes and LDSCs in the stem cell niche, research has identified other cell types which play a key role in regulating LDSC function. For example, BMMSCs differentiate into ACL cells when cocultured with ACL fibroblasts [51]; therefore, the influence of ACL fibroblasts on MSC differentiation may indicate their role in LDSC differentiation.

### 6.5. Vasculature and Innervation

As with tendon, ligament is poorly vascularised and innervated [4]; however, it is likely that these two factors still play a role within the stem cell niche. As previously discussed, one group have found CD34+ stem cells within human ACL tissue which possess all the properties found in other LDSCs [39]. This suggests that LDSCs may be derived from pericytes and the vasculature. If this is indeed the case, then the vasculature as a source of LDSCs is a vital part of the ligament stem cell niche. However, other studies have shown null expression of haematopoietic and endothelial markers in LDSCs [15, 21], suggesting the presence of two sources of LDSCs.

## 7. Therapeutic Manipulation of the Stem Cell Niche

Manipulation of stem cells* in vivo* via modulation of the stem cell niche holds therapeutic potential for the treatment of a number of conditions. Although stem cell therapies show promising results and successful outcomes, there are a number of issues associated with cell transplantation therapies. The use of autologous stem cells, and the associated isolation, culture and implantation of cells can be expensive and a long process involving invasive and painful techniques in order to extract cells from the patient. Allogeneic stem cells can be used to avoid some of these issues; however, immunosuppression may then be required which can result in problematic long-term side effects. In addition, whether autologous or allogeneic cells are used, the survival of these cells when introduced into wounds or diseased tissue is low [52].

A possible alternative to stem cell transplantation therapies is modulation of stem cells* in vivo* which can be achieved via manipulation of the stem cell niche. This can promote survival, proliferation, and differentiation of stem cells. Costly and time-consuming laboratory processes can be avoided, as well as invasive surgical techniques and the requirement for immunosuppressants. Several clinical trials are currently underway or have been recently completed which are investigating the safety and efficacy of stem cell modulation therapies. For example, the administration of a thrombopoietin mimetic to patients with severe aplastic anaemia has been shown to increase haematopoietic stem cell numbers as well as differentiation of these cells [53, 54], demonstrating the benefit of targeting secreted factors for stem cell modulation. Granulocyte/macrophage colony stimulating factor (GM-CSF) has been shown to regulate survival, proliferation, and differentiation of a number of stem cell types and when injected into chronic skin ulcers can cause modulation of the stem cell niche by increasing vascularisation [55]. This work indicates the potential advantages of targeting other cell types within the stem cell niche.

Therefore, it is possible that modulation of the ligament stem cell niche could also influence the behaviour of ligament stem cells* in vivo* and provide potential therapeutic benefits. As the quantity of research surrounding ligament stem cells increases, it may be possible to identify specific factors within their stem cell niche which can be targeted to produce a desired change in phenotype and/or function. This approach could provide a new therapeutic strategy to treat ligament injury and degeneration.

## 8. Conclusion

Although the number of studies investigating LDSCs of nondental origin is increasing, particularly in recent years, there is still very little information on many aspects of these cells. Further investigation is required into characterisation, function, and therapeutic potential of LDSCs of nondental origin. Investigation into cellular marker expression and the identification of LDSC-specific markers would be beneficial for future research in this area, as well as aiding clinical implementation. Very little research has investigated the survival and function of LDSCs* in vivo* or utilised these cells for tissue engineering strategies in animal models; therefore, the true therapeutic potential of these cells is as yet unknown and is a promising area for future research.

## Figures and Tables

**Table 1:** The expression of stem cell and tenogenic markers in LDSCs in various species. + = positive expression; − = no expression; blank = expression unknown. Information gathered from Cheng et al., 2009; Cheng et al., 2010; Kowalski et al., 2015; Shikh Alsook et al., 2015; Steinert et al., 2011; Zhang et al., 2011; our own unpublished findings.

Species	Oct-4	Nanog	SOX2	SSEA-1	SSEA-4	Nucleostemin	CD90	CD105	CD73	CD44	CD146	CD166	CD29	CD13	SCX	TNC	TNMD	CD34	CD45
Human	+	+	+		+	+	+	+	+	+	+	+	+	+		+	+	−	−
Rabbit							+			+								−	
Dog	+						+	+	+	+								−	−
Horse	+			+			+	+	+										−
